# Quantifying nitrous oxide production rates from nitrification and denitrification under various moisture conditions in agricultural soils: Laboratory study and literature synthesis

**DOI:** 10.3389/fmicb.2022.1110151

**Published:** 2023-01-12

**Authors:** Hui Wang, Zhifeng Yan, Xiaotang Ju, Xiaotong Song, Jinbo Zhang, Siliang Li, Xia Zhu-Barker

**Affiliations:** ^1^School of Earth System Science, Institute of Surface-Earth System Science, Tianjin University, Tianjin, China; ^2^Critical Zone Observatory of Bohai Coastal Region, Tianjin Key Laboratory of Earth Critical Zone Science and Sustainable Development in Bohai Rim, Tianjin University, Tianjin, China; ^3^College of Tropical Crops, Hainan University, Haikou, China; ^4^State Key Laboratory of Urban and Regional Ecology, Research Center for Eco-Environmental Sciences, Chinese Academy of Sciences, Beijing, China; ^5^School of Geography Sciences, Nanjing Normal University, Nanjing, China; ^6^Department of Soil Science, University of Wisconsin-Madison, Madison, WI, United States

**Keywords:** nitrous oxide, soil moisture, nitrification, denitrification, ^15^ N-labeled technique

## Abstract

Biogenic nitrous oxide (N_2_O) from nitrification and denitrification in agricultural soils is a major source of N_2_O in the atmosphere, and its flux changes significantly with soil moisture condition. However, the quantitative relationship between N_2_O production from different pathways (i.e., nitrification vs. denitrification) and soil moisture content remains elusive, limiting our ability of predicting future agricultural N_2_O emissions under changing environment. This study quantified N_2_O production rates from nitrification and denitrification under various soil moisture conditions using laboratory incubation combined with literature synthesis. ^15^N labeling approach was used to differentiate the N_2_O production from nitrification and denitrification under eight different soil moisture contents ranging from 40 to 120% water-filled pore space (WFPS) in the laboratory study, while 80 groups of data from 17 studies across global agricultural soils were collected in the literature synthesis. Results showed that as soil moisture increased, N_2_O production rates of nitrification and denitrification first increased and then decreased, with the peak rates occurring between 80 and 95% WFPS. By contrast, the dominant N_2_O production pathway switched from nitrification to denitrification between 60 and 70% WFPS. Furthermore, the synthetic data elucidated that moisture content was the major driver controlling the relative contributions of nitrification and denitrification to N_2_O production, while NH_4_^+^ and NO_3_^−^ concentrations mainly determined the N_2_O production rates from each pathway. The moisture treatments with broad contents and narrow gradient were required to capture the comprehensive response of soil N_2_O production rate to moisture change, and the response is essential for accurately predicting N_2_O emission from agricultural soils under climate change scenarios.

## 1. Introduction

Nitrous oxide (N_2_O) is a potent long-lived greenhouse gas, with global warming potential 296 times higher than carbon dioxide (CO_2_; Tian et al., 2020). Agricultural soil has been identified as a major source of atmospheric N_2_O, accounting for approximately 60% of the global anthropogenic N_2_O emissions (Reay et al., 2012; Cui et al., 2021). Soil moisture content is a primary regulator to control N_2_O emissions from agricultural systems (Congreves et al., 2019). Particularly, the N_2_O emissions from the soils under high moisture conditions (e.g., after rainfall or irrigation events) can constitute more than 30% of the annual emission (Trost et al., 2013; Ju and Zhang, 2017); this proportion will likely increase with the intensive use of irrigation under droughts and the increase in the frequency of heavy rainfalls, both of which were projected as a consequence of climate change (Reichstein et al., 2013; Siebert et al., 2015). However, the quantitative relationships between soil N_2_O emissions from various biological processes, including nitrification, dentification, dissimilatory nitrate reduction to ammonium (DNRA) and anaerobic ammonia oxidation, and soil moisture content remain understudied (Castellano et al., 2010; Hall et al., 2018; Li et al., 2022), impeding our ability to predict the future N_2_O emission from agricultural systems.

Nitrification and denitrification are two of the most important biological processes to produce N_2_O (Butterbach-Bahl et al., 2013), and soil moisture content substantially controls the relative contributions of these two pathways and their production rates of N_2_O (Ciarlo et al., 2007; Congreves et al., 2019). Therefore, how to accurately describe the relationships between N_2_O production rates of nitrification and denitrification and moisture content in mathematical models is crucial for estimating and predicting the N_2_O emission from soils (Yue et al., 2019). Current models, such as DNDC (Li et al., 2000) and DayCent (Parton et al., 1996), have used various types of relationships, including linear, parabolic, and exponential ones, to depict the response of N_2_O production rate to moisture change (Wang et al., 2021), regardless of the fact that the N_2_O production rates from nitrification and denitrification were theoretically expected to first increase and then decrease as moisture content increases (Davidson et al., 2000). These divergent relationships inevitably result in large uncertainty in simulating soil N_2_O emission (Gaillard et al., 2018), and accurately quantifying the relationships between N_2_O production rate and moisture content is urgently required.

Although many studies have measured the response of total N_2_O production rate to changes in moisture content (Dobbie and Smith, 2001; Schaufler et al., 2010; Cheng et al., 2014; Hall et al., 2018; Kuang et al., 2019), only a few quantified the N_2_O production rates of nitrification and denitrification under different moisture conditions (Pihlatie et al., 2004; Bateman and Baggs, 2005). In these studies, unidirectional increases in the N_2_O production rates of denitrification and nitrification were often reported as moisture increased, which contrasted with the classic hole-in-pipe model (Davidson et al., 2000). This inconsistency can be attributed to many factors such as soil physicochemical properties and measurement approaches (Liu et al., 2018; Qin et al., 2021). Among these factors, moisture treatments used in different studies should be the primary driver, since the majority of these studies adopted insufficient gradients and inadequate levels of soil moisture (Bateman and Baggs, 2005; Chen et al., 2014), which failed to capture the comprehensive change in N_2_O production rates in response to varied moisture conditions (Smith, 2017). Therefore, sufficient moisture treatments with broad range and narrow gradient are required to fill the gap between measurements and expectations.

This study hypothesizes that the production rates of N_2_O from both nitrification and denitrification first increase and then decrease as moisture content increases. We tested this hypothesis by using both laboratory incubation and literature synthesis. In the laboratory study, a ^15^N-labeled technique was applied to distinguish the nitrification and denitrification under eight moisture levels in the agricultural soils from the North China Plain. For the literature synthesis, data derived from different differentiation approaches under various moisture conditions across global agricultural soils were analyzed. The results refined the quantitative relationships between N_2_O production rate and moisture content from both nitrification and denitrification, and laid a foundation to improve the modeling of N_2_O emissions from agricultural soils.

## 2. Materials and methods

### 2.1. Site description and soil sampling

Soil samples (0–15 cm) were collected from agricultural fields in two locations: Shang Zhuang (SZ), Beijing (39°48′N, 116°28′E) and Luan Cheng (LC), Hebei (37°53′ N, 114°41′E), North China Plain, in October 2020. The annual average temperature is 12.5°C, and the annual precipitation is 500–700 mm with high variation among different years. The cropping system in this region is winter wheat-summer maize rotation. The fertilizer application rates were 280 and 600 kg N ha^−1^ year^−1^ in SZ and LC soils, respectively. Collected soils were air-dried and sieved to 2 mm. Visible roots and leaves were removed with tweezers and the soil was immediately stored at 4°C until the beginning of laboratory experiment. The soils are both classified as silt loam, with 36.1% sand, 56.4% silt, and 7.5% clay for the SZ soil and 29.2% sand, 64.1% silt, and 6.7% clay for the LC soil. For the SZ soil, pH was 7.89, bulk density was 1.02 g cm^−3^, soil organic carbon was 10.93 g kg^−1^, total N was 1.13 g kg^−1^, NH_4_^+^-N was 3.07 mg kg^−1^, and NO_3_^−^-N was 22.5 mg kg^−1^. For the LC soil, pH was 7.92, bulk density was 1.00 g cm^−3^, soil organic carbon was 19.82 g kg^−1^, total N was 2.11 g kg^−1^, NH_4_^+^-N was 2.08 mg kg^−1^, and NO_3_^−^-N was 30.49 mg kg^−1^.

### 2.2. ^15^N tracing incubation experiment

Soils (20 g oven-dry equivalent) were placed into 120 ml incubation flasks and distilled water was added to the soils to below the target moisture contents [i.e., 40, 60, 70, 80, 90, 95, 100, and 120% water-filled pore space (WFPS)]. The microcosms were then pre-incubated at 25°C for 7 days to initiate microbial activity. For each moisture content treatment, ^15^NH_4_Cl (10.08 atom%) + KNO_3_ or K^15^NO_3_ (10.16 atom%) + NH_4_Cl were applied at a rate of 50 mg NH_4_^+^-N kg^−1^ and 50 mg NO_3_^−^-N kg^−1^ after pre-incubation. To assure uniform distribution, 2 ml of ^15^N solution was applied in water solution and sprayed onto the soils to obtain the target moisture content. The experimental design and treatment application were set up as completely randomized blocks and incubated in dark for 48 h at 25°C after ^15^N application.

Each treatment was replicated three times for gas analyses, with gas samples collected at 12, 24, and 48 h. Before sampling, the flasks were flushed with ambient air using a multiport vacuum manifold, and the N_2_O concentration in the headspace was then measured. Thereafter, the flasks were immediately sealed for 12 h and N_2_O concentration was measured again. The difference between the two N_2_O concentrations was used to calculate the N_2_O production rate. The concentrations of N_2_O and CO_2_ were determined using gas chromatography (Agilent 7,890, Santa Clara, CA, United States) and the ^15^N signature of N_2_O was determined using a Thermo Finnigan MAT-253 spectrometer (Thermo Fisher Scientific, Waltham, MA, United States). Another group of flasks, also replicated three times, were used for soil sampling at 0.5, 12, 24, and 48 h after N application. Soils were extracted with 1 M KCl (20 g soil to 100 ml KCl solution), shaken for 1 h, and filtered. The concentrations of NH_4_^+^-N and NO_3_^−^-N in the extracts were measured using a continuous-flow analyzer (Skalar Analytical, Breda, Netherlands). Isotope analysis of NH_4_^+^-N and NO_3_^−^-N were performed on aliquots of the extracts using a diffusion technique (Brooks et al., 1989) and the ^15^N isotopic signature was measured by isotope ratio mass spectrometry (IRMS 20–22, Sercon, Crewe, United Kingdom).

### 2.3. Calculation

Nitrous oxide and CO_2_ fluxes (*F*, μg N kg^−1^ h^−1^ or mg C kg^−1^ h^−1^) were determined from the concentrations at each sampling time, using the background N_2_O and CO_2_ concentrations in the ambient air as the initial time point, which were calculated as follows:


(1) $$F=\frac{\rho \times \Delta c\times V\times 273}{W\times \Delta t\times \left(273+T\right)}$$

where ρ is the density of gas under standard conditions (kg m^−3^), Δ*C* is the variation in gas concentration during the flask-covering period (the units of N_2_O and CO_2_ are ppbv and ppmv, respectively), and *V* is the effective volume of a given flask (m^3^), *T* is the incubation temperature (°C), Δ*t* is the incubation time (h), and *W* is the weight of soil (oven-dried basis, kg).

The contributions of denitrification, $C_{d}$, and nitrification, $C_{n}$ to the production of N_2_O were calculated using the following equation (Stevens et al., 1997):


(2) $$C_{d}=\frac{\left(a_{N_{2}O}-a_{NH_{4}}\right)}{\left(a_{NO_{3}}-a_{NH_{4}}\right)}\;\text{with}\;a_{NO_{3}}\neq a_{NH_{4}}$$

(3) $$C_{n}=1-C_{d}$$

where *a_N2O_* is the ^15^N atom% enrichment of the N_2_O produced by both processes, and *a_NO3_* and *a_NH4_* are the ^15^N atom% enrichment of soil NO_3_^−^ and NH_4_^+^ at the time of gas sampling.

Rates of N_2_O production from nitrification (*N_2_O_n_*) and denitrification (*N_2_O_d_*) were calculated as follows:


(4) $$N_{2}O_{n}=C_{n}\times N_{2}O_{T}$$

(5) $$N_{2}O_{d}=C_{d}\times N_{2}O_{T}$$

where *N_2_O_T_* is the total N_2_O production rate from the soils, *N_2_O_T_ = N_2_O_n_ + N_2_O_d_*.

Since the concentrations and abundances of NH_4_^+^ at 48 h could not be reliably determined in most treatments, the average $C_{d}$, $C_{n}$, $N_{2}O_{n}$ and $N_{2}O_{d}$ over the first 24 h incubation were used to analyze the rates of N_2_O production from nitrification and denitrification.

### 2.4. Literature synthesis

Data on the N_2_O production rates of nitrification and denitrification were collected from published peer-reviewed journal articles. The following criteria were used for data collection: (1) incubation experiments used agricultural soils solely; (2) soil moisture metric was expressed as WFPS. Meanwhile, soil characteristics and incubation conditions, including pH, BD, clay content, SOC content, concentrations of TN, NH_4_^+^, and NO_3_^−^, incubation temperature and WFPS, were collected. GetData Graph Digitizer 2.26 was used when data were only graphically shown. The autotrophic nitrification and heterotrophic nitrification were summed and treated as nitrification during the data analysis if they were reported as individual pathways in the literature. In total, 80 groups of data from 17 studies were obtained (Supplementary Table S1).

### 2.5. Statistical analysis

All statistical analyses were evaluated by one-way analysis of variance (ANOVA) for comparisons among multiple factors and t-test for contrasts between two factors, followed by the least significant difference test at *P*<0.05. The relationships between the contributions of nitrification and denitrification to N_2_O production or their rates and the controlling factors were examined by correlation and regression analysis. All statistical analyses were carried out in SPSS v25.0 software for Windows (SPSS Inc., Chicago, United States).

## 3. Results

### 3.1. Changes in concentrations of NH_4_^+^ and NO_3_^−^ and production rate of nitrous oxide

**Figure 1:**
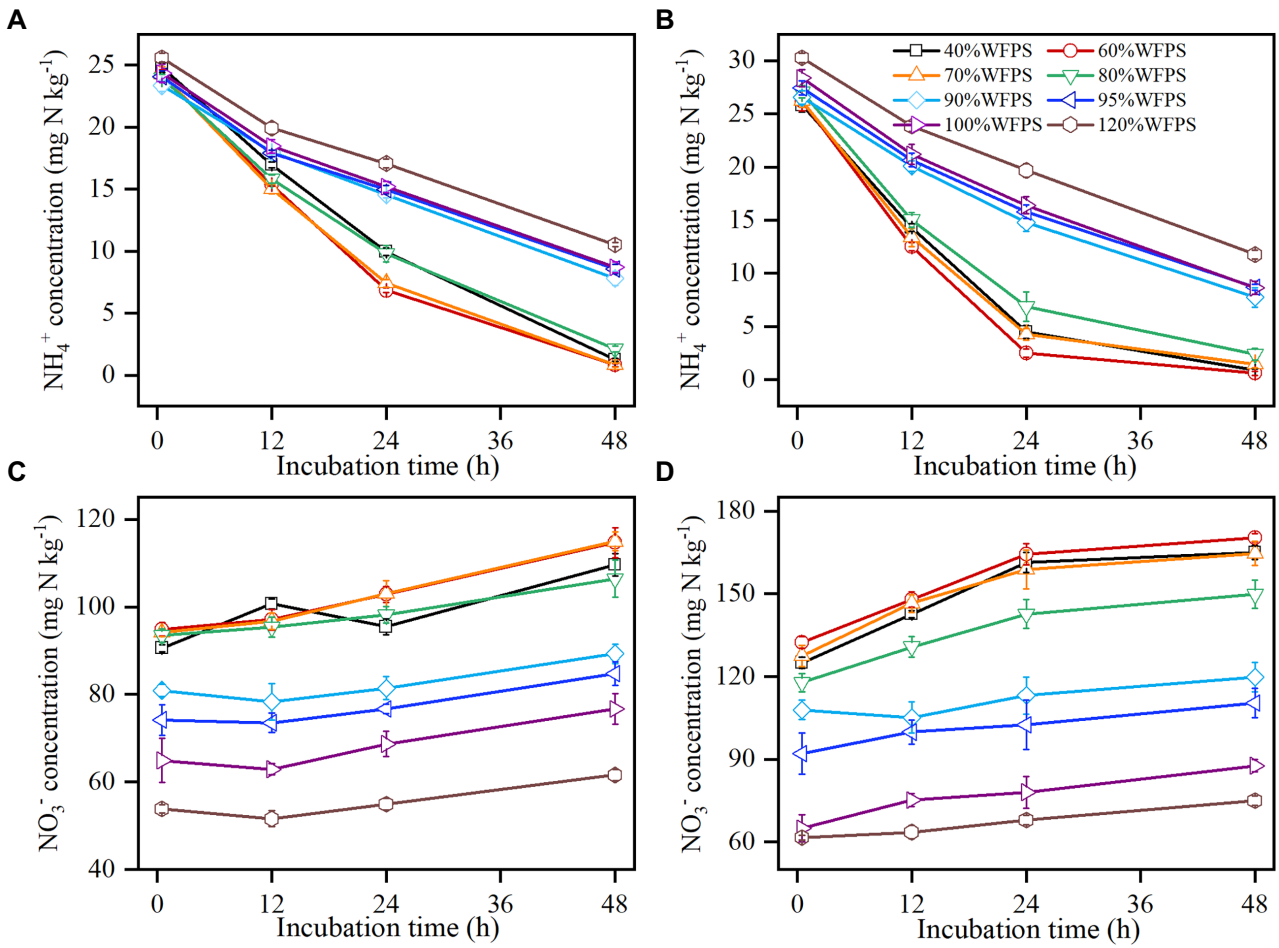
Changes in concentrations of ammonium (NH_4_^+^) and nitrate (NO_3_^−^) over 48 h of incubations in SZ **(A,C)** and LC **(B,D)** soils. Vertical bars are standard deviations of the means (*n* = 6).

The concentration of soil NH_4_^+^ decreased over the incubation course in all moisture treatments (Figures 1A,B). For both SZ and LC soils, the declining rates of NH_4_^+^ over the first 24 h were nearly twice larger in the treatments of WFPS ≤80% than in the treatments of WFPS ≥90%. After the first 24 h, the declining rate slowed down clearly when WFPS ≤80%, especially for the LC soil (Figure 1B), while it nearly kept constant under WFPS ≥90%. Among all the WFPS treatments, the largest consumption rate of NH_4_^+^ occurred at 60% WFPS for both SZ and LC soils.

The concentration of soil NO_3_^−^ increased as NH_4_^+^ was nitrified (Figures 1C,D). In correspondence to the changes in NH_4_^+^ concentration, NO_3_^−^ concentration increased faster when WFPS ≤80% than when WFPS ≥90%, especially for the LC soil during the first 24 h. The initial NO_3_^−^ concentration exhibited large variances for different moisture contents, since nitrification increased NO_3_^−^ concentration under low moisture content while denitrification reduced NO_3_^−^ concentration under high moisture during the pre-incubation period. As the initial NO_3_^−^ concentration markedly reduced as WFPS increased, the NO_3_^−^ concentration varied largely at the end of incubation especially for the LC soil, changing from 170.3 to 75.0 mg N kg^−1^ as WFPS increased from 60 to 120%.

**Figure 2:**
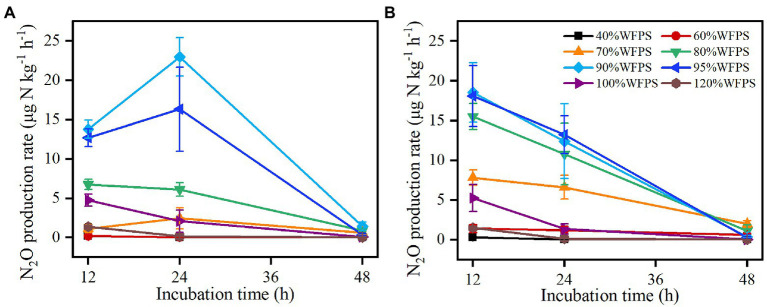
Changes in N_2_O production rate over 48 h of incubations from SZ **(A)** and LC **(B)** soils. Vertical bars are the standard deviations of the means (*n* = 6).

The N_2_O production rate changed substantially with moisture content and time (Figure 2). At the beginning of incubation, high N_2_O production rates (> 5 μg N kg^−1^ h^−1^) occurred under 80% ≤ WFPS ≤100% in the SZ soil and under 70% ≤ WFPS ≤100% in the LC soil, whereas the rates remained low under the lower or higher moisture conditions. As the incubation proceeded, the N_2_O production rate first increased and then decreased under the intermediate moisture conditions (e.g., WFPS = 70, 90, and 95%) in the SZ soil, but consistently reduced under all moisture conditions in the LC soil. Finally, the N_2_O production rates declined to below 5 μg N kg^−1^ h^−1^ under all moisture contents for both soils at the end of incubation. By contrast, CO_2_ production rates were higher at WFPS ≥90% than at WFPS <90% for both soils, except for 95% WFPS in the LC soil (*P*<0.05; Supplementary Figure S1).

### 3.2. Nitrous oxide production from nitrification and denitrification

**Figure 3:**
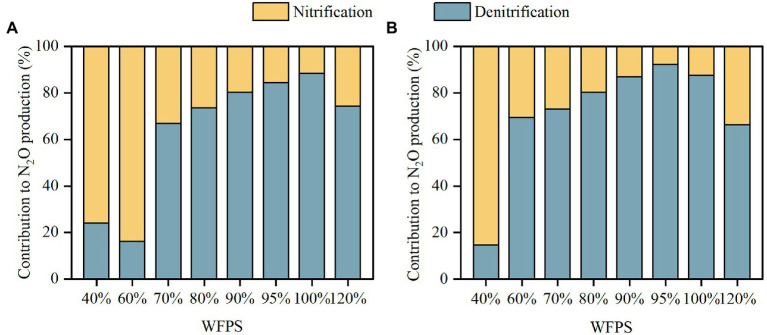
The contributions of nitrification and denitrification to N_2_O production in SZ **(A)** and LC **(B)** soils.

The ^15^N enrichment of N_2_O remained between the ^15^N enrichments of NH_4_^+^ and NO_3_^−^ during the first 24 h, illustrating that N_2_O was derived from both nitrification and denitrification (Supplementary Figure S2). The average contribution of denitrification to N_2_O production, $C_{d}$, increased with moisture content in the SZ and LC soils up to 100 and 95% WFPS, respectively, after which $C_{d}$ declined significantly (Figure 3). In both soils, nitrification was the main pathway producing N_2_O under low moisture conditions while denitrification dominated N_2_O production under high moisture conditions, with the threshold occurred at 70 and 60% WFPS for the SZ and LC soils, respectively. Denitrification contributed more than 65% of the total N_2_O production when WFPS ≥70%, and this percentage promoted as the incubation proceeded (Supplementary Table S2).

**Figure 4:**
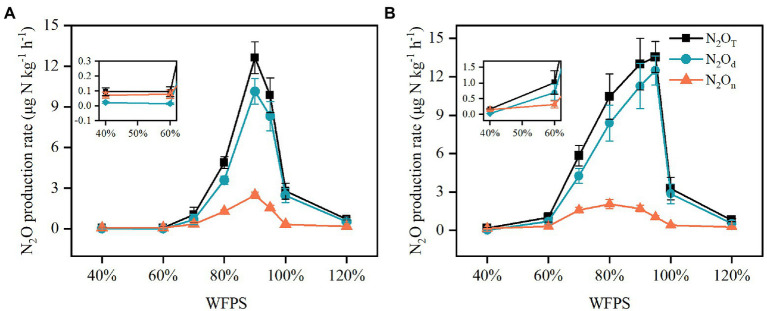
The N_2_O production rates derived from nitrification (*N_2_O_n_*), denitrification (*N_2_O_d_*) and the combined processes (*N_2_O_T_*) in the SZ **(A)** and LC **(B)** soils under different WFPS.

Nitrous oxide production rates derived from nitrification (*N_2_O_n_*), denitrification (*N_2_O_d_*) and the combined processes (*N_2_O_T_*) responded to moisture change in a pattern similar to Gaussian function in both SZ and LC soils (Figure 4). As moisture increased, the *N_2_O_n_* increased slowly, reaching peaks around 2.5 μg N kg^−1^ h^−1^ in both SZ and LC soils, while the *N_2_O_d_* increased steeply, reaching peaks of 10.1 and 12.5 μg N kg^−1^ h^−1^ in the SZ and LC soils, respectively. Correspondingly, the optimal WFPS with respect to the peak rates were the same for the nitrification and denitrification processes (90% WFPS) in the SZ soil, but diverged for the two pathways (80 and 95% WFPS, respectively) in the LC soil. The N_2_O production rates remained below 3 μg N kg^−1^ h^−1^ under either low or flooded moisture condition.

### 3.3. Literature synthesis: Nitrous oxide production from nitrification and denitrification across agricultural soils

**Figure 5:**
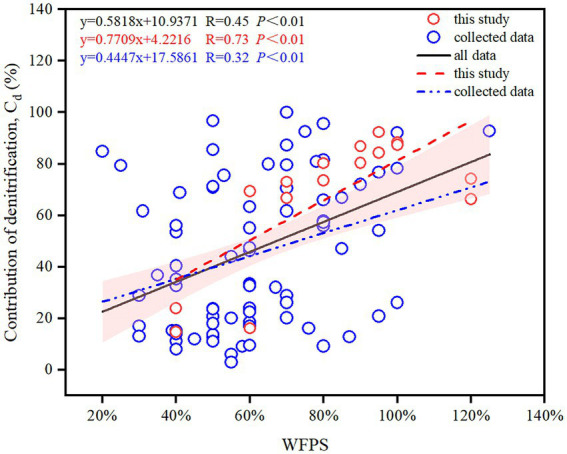
Changes in the contribution of denitrification to N_2_O production ($C_{d}$) with WFPS across global agricultural soils. The shaded region represents the 95% confidence interval for all data.

By synthesizing literature data across global agricultural soils, moisture (WFPS) and incubation temperature (T) were found to be the most significant factors controlling the contributions of nitrification and denitrification to N_2_O production (Table 1), with WFPS exerting a stronger correlation (R = 0.45) than T (R = 0.37; Figure 5; Supplementary Figure S3). Compared with the literature data (R = 0.36), the measured data in this study exhibited a stronger positive correlation between $C_{d}$ and WFPS (R = 0.73; Figure 5). Furthermore, a stronger correlation between $C_{d}$ and WFPS occurred in alkaline soils than in acidic soils (Supplementary Figure S4A). Similarly, compared with carbon-rich soils with SOC ≥ 4%, mineral soils with SOC < 4% showed a stronger correlation (Supplementary Figure S4B).

**Table 1:** Correlations between the contribution of denitrification ($C_{d}$) and soil properties as well as environmental conditions, which include soil pH, bulk density (BD), clay content, soil organic carbon (SOC), total nitrogen (TN) concentrations, NH_4_^+^ and NO_3_^−^ concentrations, incubation temperature (T) and water-filled pore space (WFPS), across agricultural soils.

		pH	BD	Clay	SOC	TN	NH_4_^+^	NO_3_^−^	T	WFPS
$C_{d}$	R	0.076	−0.147	−0.022	0.112	0.088	0.198	−0.249	0.369	0.446
	*P*	0.462	0.335	0.858	0.340	0.414	0.187	0.096	<0.01	<0.01
	n	96	45	70	75	89	46	46	94	96

**Figure 6:**
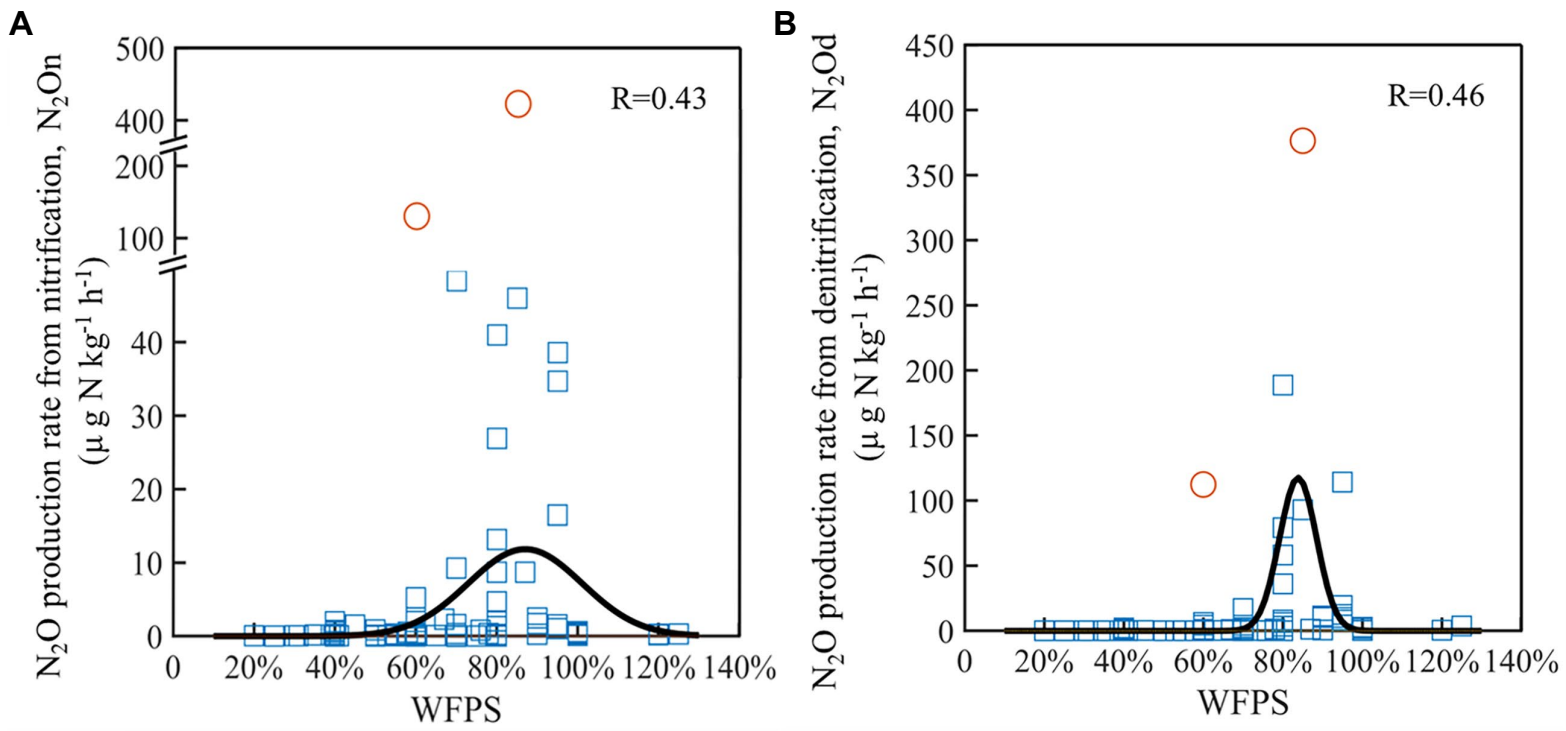
Changes in N_2_O production rates from nitrification **(A)** and denitrification **(B)** under different WFPS across agricultural soils. The black lines were the fitted curves using Gaussian function after excluding the abnormal values (the circles).

Based on the literature synthesis, the *N_2_O_n_* and *N_2_O_d_* generally first increased and then decreased as WFPS increased (Figure 6). The relationships between the N_2_O production rates of nitrification and denitrification and WFPS were fitted by Gaussian function. Compared with nitrification (Figure 6A), denitrification (Figure 6B) showed a smaller standard deviation, 5% vs. 14%, and a higher maximum rate, 106 vs. 12 μg N kg^−1^ h^−1^, though both of their peak rates occurred at around 85% WFPS. The correlations between N_2_O production rates and various soil properties were also analyzed (Supplementary Table S3). The results indicated that NH_4_^+^ and NO_3_^−^ concentrations were the most powerful drivers to explain the changes in *N_2_O_n_* and *N_2_O_d_*. Both *N_2_O_n_* and *N_2_O_d_* increased positively with the increases in NH_4_^+^ (*P*<0.05; Supplementary Figures S5A,C) and NO_3_^−^ concentrations (*P*<0.01; Supplementary Figures S5B,D), though the variances of rates were large as the concentrations were high.

## 4. Discussion

### 4.1. Contributions of nitrification and denitrification to nitrous oxide production

Both laboratory incubation and literature synthesis showed that nitrification and denitrification dominated N_2_O production under low and high moisture conditions, respectively. Under high moisture conditions as soil oxygen availability was constrained, denitrification outcompeted nitrification as the main source of N_2_O production (Smith, 2017; Song et al., 2019; Chang et al., 2022), which was aligned with other experiments (Pihlatie et al., 2004; Friedl et al., 2021). The dominant pathway of N_2_O production switched between 60 and 70% WFPS (Figure 5), depending on soil properties and climatic conditions. For instance, the thresholds for SZ and LC soil were 70 and 60% WFPS (Figure 3), respectively. This is because the SOC content was higher in the LC soil (19.82 g kg^−1^) than in the SZ soil (10.93 g kg^−1^), stimulating N_2_O production by promoting denitrification process (Ruser et al., 2006; Chantigny et al., 2013). Besides, the N_2_O production rate in the LC soil (1.02 μg N kg^−1^ h^−1^) was almost 10 times that in SZ soil (0.1 μg N kg^−1^ h^−1^) under 60% WFPS, further indicating the dominating effects of denitrification in the N_2_O production in the LC soil. The literature synthesis also confirmed that large SOC content increased the contribution of denitrification to N_2_O production under relatively low soil moisture content (Supplementary Figure S4B). Besides SOC, other factors such as BD, NH_4_^+^ and NO_3_^−^ concentrations, and especially incubation temperature, also modulated the contributions of nitrification and denitrification to N_2_O production (Table 1), which might explain why the contribution proportions between nitrification and denitrification varied significantly among different soils even though the soil moisture status were similar (Figure 5).

Accurately determining the contributions of nitrification and denitrification to N_2_O production is crucial to evaluate N_2_O emissions from agricultural soils (Zhu et al., 2013). Currently, different approaches were used to quantify these contributions, including ^15^N site preference (Thilakarathna and Hernandez-Ramirez, 2021), acetylene inhibition (Pihlatie et al., 2004), and ^15^N tracing techniques (Friedl et al., 2021). The applications of these approaches often caused large discrepancies in quantifying $C_{d}$ and $C_{n}$ under different moisture conditions (Butterbach-Bahl et al., 2013), and likely resulted in different contribution proportions even though the experimental setup and the operating conditions were the same (Zhu et al., 2013). Therefore, a careful comparison among different approaches and developing a guideline or protocol for using these approaches merit further investigations. Although certain factors such as pH value and N concentrations exerted insignificant impacts on the contribution of different pathways to N_2_O production (Table 1), their integrative impacts remain unclear (Hu et al., 2015). In addition, factors such as moisture and temperature, often changed synchronously in fields (Song et al., 2018), and studying their integrative impacts will significantly improve our understanding of N_2_O emission dynamics and facilitate N_2_O abatement (Mathieu et al., 2006).

### 4.2. Nitrous oxide production rates of nitrification and denitrification

Both laboratory study and literature synthesis validated the hypothesis that the rates of N_2_O production from both nitrification and denitrification first increased and then decreased as soil moisture increased (Figures 4, 6). The relationships between N_2_O production rate and moisture content followed the classic hole-in-pipe model (Davidson et al., 2000), though the rates changed with soil properties (Figure 4). For instance, the LC soil produced generally larger *N_2_O_d_* than the SZ soil, since it contained more NO_3_^−^ and SOC, which stimulated N_2_O production from denitrification under high moisture content (Smith, 2017). By comparison, the two soils exhibited approximate *N_2_O_n_* due to the similar NH_4_^+^ concentrations. The literature synthesis further confirmed that NO_3_^−^ and NH_4_^+^ were the two most important factors to determine N_2_O production rates (Supplementary Table S3). Interestingly, NO_3_^−^ concentration was the most powerful driver to explain the changes in N_2_O derived from nitrification, although its explaining power was close to that of NH_4_^+^ concentration. This result might be caused by the large N_2_O production rates from nitrification under high NO_3_^−^ concentrations and large soil moisture contents (Supplementary Figure S5) and warrant further investigations. However, the rates of *N_2_O_d_* and *N_2_O_n_* depended on not only the above factors but also moisture content, and their interactions control N_2_O emission from soils (Zhu et al., 2013). Therefore, higher substrate concentration unnecessarily resulted in larger N_2_O emissions, as being observed in many laboratory and field experiments (Senbayram et al., 2012; Liu et al., 2018).

In contrast to the first increased and then decreased N_2_O production rates in response to increase in soil moisture content from the laboratory incubation in this study, the studies in the collected literatures presented divergent consequences among different experiments (Supplementary Table S1). Among the 17 collected studies, as moisture increased, only five reported a decline in N_2_O production rate for nitrification and no study found a decline for denitrification. The underrepresented decline in the rates can be mainly attributed to the insufficient gradients and inadequate levels of moisture content applied in these studies, which commonly used soil moisture containing less than four levels and below 90% WFPS (Supplementary Table S1). Such sparse moisture levels likely did not capture the inflection point of N_2_O production rate (Barton et al., 2015), while the low moisture condition might not be adequate to capture the turning point (Bateman and Baggs, 2005; Liu et al., 2016). Therefore, N_2_O emission under relatively high moisture conditions with sufficient moisture treatments deserves further investigations. The interactions of soil moisture with other factors such as SOC content (Qin et al., 2017), nutrient availability (Senbayram et al., 2012), and pH value (Zhang et al., 2015) together determine the relationship between N_2_O emission rates and moisture contents (Zhu et al., 2020).

### 4.3. Implications and looking forward

Both laboratory study and literature synthesis illustrated that N_2_O emissions declined as moisture content exceeded certain threshold. Current models using linear or exponential relationships between N_2_O production rate and moisture content could significantly overestimate N_2_O emissions from agricultural systems (Yue et al., 2019; Wang et al., 2021), especially as the intensive irrigation and extreme rainfall are projected to increase under climate change scenarios (Smith et al., 2017). Therefore, comprehensive relationships that can capture the first increased and then decreased N_2_O production rates in response to elevated soil moisture content are required. However, the large variances in N_2_O production rates of both nitrification and denitrification among different studies induce great challenges to develop such a relationship. One potential breakthrough can be to quantify this relationship for different types of soils by incorporating intense moisture treatments similar to this study. Meanwhile, additional experiments are required to quantify the impacts of other key factors, such as temperature, NO_3_^−^ and NH_4_^+^ concentrations and their interactions, on the relationship. Once sufficient data measured using the same experimental protocol are collected, it will be possible to derive quantitative relationships between N_2_O production rate and moisture content across different soils by using a general function, such as Gaussian function, with parameters depending on key edaphic and climatic drivers (Yan et al., 2018).

## 5. Conclusion

This study quantified the response of soil N_2_O production rates from nitrification and denitrification to changes in a broad range of moisture contents using both laboratory study and literature synthesis. The results showed that the N_2_O production rates of nitrification and denitrification first increased and then decreased as moisture increased for both particular and global agricultural soils, following the classic hole-in-pipe model. The inflection points of moisture content, under which the N_2_O production rate maximized, for the two pathways occurred between 80 and 95% WFPS, which value depended on incubation temperature and soil properties. By contrast, the switching point of soil moisture from nitrification-dominating to denitrification-dominating occurred between 60 and 70% WFPS. The unidirectional increase in N_2_O production rates reported in most literatures should be attributed to the insufficient gradients and inadequate levels of moisture content applied in the incubation experiments, and moisture treatments containing broad moisture contents with narrow gradient are required to obtain the comprehensive relationship between soil N_2_O production rate and moisture content, which is crucial to accurately predict future N_2_O emission from global agricultural soils in response to climate change.

## Data availability statement

The raw data supporting the conclusions of this article will be made available by the authors, without undue reservation.

## Author contributions

HW conducted the experiments and wrote the first draft. ZY guided the experiments and completed the final draft. XZ-B, XJ, XS, JZ, and SL helped to improve the manuscript. All authors contributed to the article and approved the submitted version.

## Funding

This work was financially supported by the National Key Research and development Program of China (No. 2022YFF1301002), National Natural Science Foundation of China (No. 42077009), and Haihe Laboratory of Sustainable Chemical Transformations.

## Conflict of interest

The authors declare that the research was conducted in the absence of any commercial or financial relationships that could be construed as a potential conflict of interest.

## Publisher’s note

All claims expressed in this article are solely those of the authors and do not necessarily represent those of their affiliated organizations, or those of the publisher, the editors and the reviewers. Any product that may be evaluated in this article, or claim that may be made by its manufacturer, is not guaranteed or endorsed by the publisher.
