# Supplementary material for: Quantifying nitrous oxide production rates from nitrification and denitrification under various moisture conditions in agricultural soils: Laboratory study and literature synthesis
Source: Front Microbiol. 2023 Jan 12;13:1110151. doi: 10.3389/fmicb.2022.1110151 (PMC9877343; doi:10.3389/fmicb.2022.1110151)
Supplement: Supplementary file 1 [file Data_Sheet_1.docx]

**Supplementary materials for**

**Quantifying N_2_O Production Rates from Nitrification and Denitrification under Various Moisture Conditions in Agricultural Soils: Laboratory Study and Literature Synthesis**

## Supplementary Tables

**Table S1** Summary of contributions and rates of N_2_O production from nitrification and denitrification based on the collected data from global agricultural soils and the measured data from this study. The soil properties and environmental variables include pH, bulk density (BD), clay content, soil organic carbon (SOC), total nitrogen (TN) concentrations, NH_4_^+^ concentrations, NO_3_^-^concentrations, incubation temperature (T) and water-filled pore space (WFPS)

**Table S2** The contribution of nitrification ($\text{C}_{\text{n}}$) and denitrification ($\text{C}_{\text{d}}$) to N_2_O production under different WFPS

**Table S3** Correlations between the N_2_On/N_2_Od and soil properties

## Supplementary Figures

**Fig. S1** CO_2_ production rates from the SZ and LC soils under different WFPS over an incubation time of 24 hours. Different lower case letters denote significant differences between SZ and LC soil according to *t*-test, and different capital letters denote significant differences among different WFPS according to one-way analysis of variance (ANOVA) (*P*< 0.05)

**Fig. S2** ^15^N enrichments of NH_4_^+^, NO_3_^-^, and N_2_O in SZ **(A, B)** and LC **(C, D)** soils under ^15^NH_4_^+^-labeled **(A, C)** and ^15^NO_3_^-^-labeled **(B, D)** treatments

**Fig. S3** Contribution of denitrification to N_2_O production ($\text{C}_{\text{d}}$) with incubation temperature (T) across global agricultural soils. The shaded region represents the 95% confidence interval for the collected and measured data

**Fig. S4** Contribution of denitrification to N_2_O production ($\text{C}_{\text{d}}$) with WFPS across global agricultural soils. Different colors denote different pH values **(A)** and SOC contents **(B)**

**Fig. S5** Changes in the *N_2_On* with NH_4_^+^-N **(A)** and NO_3_^-^-N **(B)** and changes in the *N_2_On* with NH_4_^+^-N **(C)** and NO_3_^-^-N **(D)**. The shaded region represents the 95% confidence interval for the collected and measured data

**Table S1** Summary of contributions and rates of N_2_O production from nitrification and denitrification based on the collected data from global agricultural soils and the measured data from this study. The soil properties and environmental variables include pH, bulk density (BD), clay content, soil organic carbon (SOC), total nitrogen (TN) concentrations, NH_4_^+^ concentrations, NO_3_^-^concentrations, incubation temperature (T) and water-filled pore space (WFPS)

| Country | pH | BD | Clay  (%) | SOC  (g kg^-1^) | TN  (g kg^-1^) | NH_4_^+^ (mg N kg^-1^) | $\text{NO}_{\text{3}}^{\text{-}}$ (mg N kg^-1^) | N amendment | | Separation method | | T (℃) | | WFPS (%) | | $\text{C}_{\text{n}}$ (%) | | $\text{C}_{\text{d}}$ (%) | | N_2_On  (μg N kg^-1^h-^1^) | | N_2_Od  (μg N kg^-1^h-^1^) | Reference |  |
| --- | --- | --- | --- | --- | --- | --- | --- | --- | --- | --- | --- | --- | --- | --- | --- | --- | --- | --- | --- | --- | --- | --- | --- | --- |
| Britain | 7.1 | 1.23 | 15 |  | 2 |  |  | $\text{NH}_{\text{4}}^{\text{+}}$: 710 mg N kg^-1^  $\text{NO}_{\text{3}}^{\text{-}}$: 710 mg N kg^-1^ | | ^15^N+C_2_H_2_ | | 21 | | 20 | | 15.3 | | 84.8 | | 0.018 | | 0.102 | (Bateman and Baggs, 2005) |  |
|  | 7.1 | 1.23 | 15 |  | 2 |  |  |  |  |  |  | 21 | | 35 | | 63.3 | | 36.7 | | 0.133 | | 0.077 |  |  |
|  | 7.1 | 1.23 | 15 |  | 2 |  |  |  |  |  |  | 21 | | 50 | | 76.1 | | 23.9 | | 0.145 | | 0.045 |  |  |
|  | 7.1 | 1.23 | 15 |  | 2 |  |  |  |  |  |  | 21 | | 60 | | 81.5 | | 18.5 | | 0.269 | | 0.061 |  |  |
|  | 7.1 | 1.23 | 15 |  | 2 |  |  |  |  |  |  | 21 | | 70 | | 0 | | 100 | | 0.000 | | 1.940 |  |  |
| China | 5.9 | 1.03 | 20 | 27.5 | 2.2 | 6.7 | 30.7 | $\text{NH}_{\text{4}}^{\text{+}}$: 50 mg N kg^-1^  $\text{NO}_{\text{3}}^{\text{-}}$: 50 mg N kg^-1^ | | ^15^N | | 25 | | 30 | | 71.2 | | 28.8 | | 0.050 | | 0.020 | (Chen et al., 2014) |  |
|  | 5.9 | 1.03 | 20 | 27.5 | 2.2 | 6.7 | 30.7 |  |  |  |  | 25 | | 50 | | 79.3 | | 20.7 | | 0.066 | | 0.017 |  |  |
|  | 5.9 | 1.03 | 20 | 27.5 | 2.2 | 6.7 | 30.7 |  |  |  |  | 25 | | 70 | | 71.1 | | 28.9 | | 0.064 | | 0.026 |  |  |
| Australia | 4.5 |  | 19 | 52 | 5.2 | 13 | 93 | $\text{NH}_{\text{4}}^{\text{+}}$: 100 mg N kg^-1^  $\text{NO}_{\text{3}}^{\text{-}}$: 50 mg N kg^-1^ | | ^15^N | | 25 | | 50 | | 87.2 | | 12.8 | | 0.061 | | 0.009 | (Liu et al., 2016a) |  |
|  | 4.5 |  | 19 | 52 | 5.2 | 13 | 93 |  |  |  |  | 25 | | 70 | | 79.8 | | 20.2 | | 9.249 | | 2.341 |  |  |
|  | 4.5 |  | 19 | 52 | 5.2 | 13 | 93 |  |  |  |  | 25 | | 85 | | 52.9 | | 47.1 | | 422.72 | | 376.38 |  |  |
|  | 4.5 |  | 19 | 52 | 5.2 | 13 | 93 |  |  |  |  | 35 | | 50 | | 86.3 | | 13.7 | | 0.854 | | 0.136 |  |  |
|  | 4.5 |  | 19 | 52 | 5.2 | 13 | 93 |  |  |  |  | 35 | | 70 | | 73.9 | | 26.1 | | 48.360 | | 17.080 |  |  |
|  | 4.5 |  | 19 | 52 | 5.2 | 13 | 93 |  |  |  |  | 35 | | 85 | | 33.1 | | 66.9 | | 45.976 | | 92.924 |  |  |
| China | 7.87 |  |  | 15.4 |  | 2.33 | 6.33 | $\text{NH}_{\text{4}}^{\text{+}}$: 100 mg N kg^-1^ | | stable isotope+ C_2_H_2_ | | 25 | | 67 | | 67.93 | | 32.07 | | 2.323 | | 1.097 | (Zheng et al., 2017) |  |
|  | 7.87 |  |  | 15.4 |  | 2.33 | 6.33 |  |  |  |  | 25 | | 80 | | 42.35 | | 57.65 | | 4.675 | | 6.365 |  |  |
|  | 7.87 |  |  | 15.4 |  | 2.33 | 6.33 |  |  |  |  | 25 | | 95 | | 79.16 | | 20.84 | | 38.591 | | 10.160 |  |  |
| Finland | 5.8 | 0.53 |  |  | 11 |  |  |  | | C_2_H_2_ | | 21 | | 60 | | 76.0 | | 24.0 | | 0.003 | | 0.001 | (Pihlatie et al., 2004) |  |
|  | 5.8 | 0.53 |  |  | 11 |  |  |  |  |  |  | 21 | | 80 | | 42.2 | | 57.8 | | 0.003 | | 0.003 |  |  |
|  | 5.8 | 0.53 |  |  | 11 |  |  |  |  |  |  | 21 | | 100 | | 21.7 | | 78.3 | | 0.128 | | 0.462 |  |  |
|  | 6.0 | 1.1 | 9.7 |  | 1.6 |  |  |  |  |  |  | 21 | | 60 | | 77.5 | | 22.5 | | 0.002 | | 0.001 |  |  |
|  | 6.0 | 1.1 | 9.7 |  | 1.6 |  |  |  |  |  |  | 21 | | 80 | | 44.1 | | 55.9 | | 0.101 | | 0.129 |  |  |
|  | 6.0 | 1.1 | 9.7 |  | 1.6 |  |  |  |  |  |  | 21 | | 100 | | 73.9 | | 26.1 | | 0.613 | | 0.217 |  |  |
|  | 6.5 | 1.1 | 57.3 |  | 2.2 |  |  |  |  |  |  | 21 | | 40 | | 67.4 | | 32.6 | | 0.001 | | 0.000 |  |  |
|  | 6.5 | 1.1 | 57.3 |  | 2.2 |  |  |  |  |  |  | 21 | | 60 | | 66.5 | | 33.5 | | 0.001 | | 0.000 |  |  |
| Australia | 6.3 |  |  | 42 | 3.6 |  |  | $\text{NH}_{\text{4}}^{\text{+}}$: 35 mg N kg^-1^  $\text{NO}_{\text{3}}^{\text{-}}$: 35 mg N kg^-1^ | | ^15^N | | 25 | | 40 | | 46.5 | | 53.5 | | 1.958 | | 2.252 | (Friedl et al., 2021) |  |
|  | 6.3 |  |  | 42 | 3.6 |  |  |  |  |  |  | 25 | | 60 | | 44.9 | | 55.1 | | 5.231 | | 6.419 |  |  |
|  | 6.3 |  |  | 42 | 3.6 |  |  |  |  |  |  | 25 | | 80 | | 34.1 | | 65.9 | | 40.985 | | 79.205 |  |  |
|  | 6.3 |  |  | 42 | 3.6 |  |  |  |  |  |  | 25 | | 95 | | 23.3 | | 76.7 | | 34.698 | | 114.222 |  |  |
|  | 6.1 |  |  | 49 | 5 |  |  |  |  |  |  | 25 | | 40 | | 44.0 | | 56.0 | | 0.770 | | 0.980 |  |  |
|  | 6.1 |  |  | 49 | 5 |  |  |  |  |  |  | 25 | | 60 | | 36.7 | | 63.3 | | 2.499 | | 4.311 |  |  |
|  | 6.1 |  |  | 49 | 5 |  |  |  |  |  |  | 25 | | 80 | | 18.4 | | 81.6 | | 13.138 | | 58.262 |  |  |
|  | 6.1 |  |  | 49 | 5 |  |  |  |  |  |  | 25 | | 95 | | 45.9 | | 54.1 | | 16.487 | | 19.433 |  |  |
|  | 5.9 |  |  | 41 | 4 |  |  |  |  |  |  | 25 | | 40 | | 59.6 | | 40.4 | | 0.584 | | 0.396 |  |  |
|  | 5.9 |  |  | 41 | 4 |  |  |  |  |  |  | 25 | | 60 | | 67.4 | | 32.6 | | 1.982 | | 0.958 |  |  |
|  | 5.9 |  |  | 41 | 4 |  |  |  |  |  |  | 25 | | 80 | | 42.7 | | 57.3 | | 26.918 | | 36.122 |  |  |
| Costa Rica | 4.7 | 0.93 | 36.8 |  | 3.7 |  |  | $\text{NH}_{\text{4}}^{\text{+}}$: 270 mg N kg^-1^  $\text{NO}_{\text{3}}^{\text{-}}$: 270 mg N kg^-1^ | | C_2_H_2_ | | 21 | | 39 | | 84.7 | | 15.3 | | 0.169 | | 0.031 | (Hergoualc’h et al., 2007) |  |
|  | 4.7 | 0.93 | 36.8 |  | 3.7 |  |  |  |  |  |  | 21 | | 58 | | 90.9 | | 9.1 | | 0.554 | | 0.056 |  |  |
|  | 4.7 | 0.93 | 36.8 |  | 3.7 |  |  |  |  |  |  | 21 | | 76 | | 83.9 | | 16.1 | | 0.805 | | 0.155 |  |  |
|  | 4.7 | 0.93 | 36.8 |  | 3.7 |  |  |  |  |  |  | 21 | | 87 | | 87.1 | | 12.9 | | 8.693 | | 1.287 |  |  |
| China | 5.06 |  | 31.13 | 34.7 | 2.22 |  |  | $\text{NH}_{\text{4}}^{\text{+}}$: 710 mg N kg^-1^  $\text{NO}_{\text{3}}^{\text{-}}$: 710 mg N kg^-1^ | | ^15^N | | 25 | | 25 | | 20.69 | | 79.31 | | 0.001 | | 0.002 | (Qin et al., 2021) |  |
|  | 5.06 |  | 31.13 | 34.7 | 2.22 |  |  |  |  |  |  | 25 | | 50 | | 14.45 | | 85.55 | | 0.001 | | 0.003 |  |  |
|  | 5.06 |  | 31.13 | 34.7 | 2.22 |  |  |  |  |  |  | 25 | | 75 | | 7.51 | | 92.49 | | 0.030 | | 0.370 |  |  |
|  | 5.06 |  | 31.13 | 34.7 | 2.22 |  |  |  |  |  |  | 25 | | 100 | | 7.95 | | 92.05 | | 0.177 | | 2.053 |  |  |
|  | 5.06 |  | 31.13 | 34.7 | 2.22 |  |  |  |  |  |  | 25 | | 125 | | 7.28 | | 92.72 | | 0.299 | | 3.811 |  |  |
| China | 8.09 | 1.47 |  | 7.75 | 0.78 | 1.70 | 10.55 | $\text{NH}_{\text{4}}^{\text{+}}$: 50 mg N kg^-1^  $\text{NO}_{\text{3}}^{\text{-}}$: 50 mg N kg^-1^ | | ^15^N | | 25 | | 40 | | 88.82 | | 11.18 | | 0.204 | | 0.026 | (Liao et al., 2021) |  |
|  | 8.09 | 1.47 |  | 7.75 | 0.78 | 1.70 | 10.55 |  |  |  |  | 25 | | 60 | | 90.43 | | 9.57 | | 0.226 | | 0.024 |  |  |
|  | 8.09 | 1.47 |  | 7.75 | 0.78 | 1.70 | 10.55 |  |  |  |  | 25 | | 80 | | 90.83 | | 9.17 | | 0.236 | | 0.024 |  |  |
| Canada | 6.2 | 1.25 | 42 | 45 | 3.9 |  |  | Urea:  62.34 mg N kg^-1^ | | δ^15^N–N_2_O site preference | | 20 | | 31 | | 38.3 | | 61.7 | | 0.038 | | 0.062 | (Thilakarathna and Hernandez-Ramirez, 2021) |  |
|  | 6.2 | 1.25 | 42 | 45 | 3.9 |  |  |  |  |  |  | 20 | | 41 | | 31.1 | | 68.9 | | 0.062 | | 0.138 |  |  |
|  | 6.2 | 1.25 | 42 | 45 | 3.9 |  |  |  |  |  |  | 20 | | 53 | | 24.5 | | 75.5 | | 0.056 | | 0.174 |  |  |
|  | 6.2 | 1.25 | 42 | 45 | 3.9 |  |  |  |  |  |  | 20 | | 65 | | 20.1 | | 79.9 | | 0.076 | | 0.304 |  |  |
|  | 6.2 | 1.25 | 42 | 45 | 3.9 |  |  |  |  |  |  | 20 | | 78 | | 19.1 | | 80.9 | | 0.351 | | 1.489 |  |  |
| Spain | 7.9 | 1.20 | 28 | 8.4 |  |  |  | Urea: 120 kg N ha^-1^  $\text{NO}_{\text{3}}^{\text{-}}$ 50 kg N ha^-1^ | | C_2_H_2_ | | - | | 60 | | 53.7 | | 46.3 | | 130.325 | | 112.365 | (Sanz-Cobena et al., 2014) |  |
|  | 7.9 | 1.20 | 28 | 8.4 |  |  |  |  |  |  |  | - | | 80 | | 4.4 | | 95.6 | | 8.693 | | 188.867 |  |  |
| Australia | 5.5 |  | 8 | 46 | 5 | 12.30 | 6.90 | $\text{NH}_{\text{4}}^{\text{+}}$: 50 mg N kg^-1^  $\text{NO}_{\text{3}}^{\text{-}}$: 50 mg N kg^-1^ | | ^15^N+C_2_H_2_ | | 20 | | 60 | | 52.5 | | 47.5 | | 0.079 | | 0.071 | (Lan et al., 2018) |  |
|  | 6.0 |  | 11 | 59 | 6 | 12.2 | 14.9 |  |  |  |  | 20 | | 60 | | 67.3 | | 32.7 | | 0.108 | | 0.052 |  |  |
| China | 8.0 | 1.3 | 16 | 23.9 | 1.2 | 0.8 | 35.5 | $\text{NH}_{\text{4}}^{\text{+}}$: 56 mg N kg^-1^  $\text{NO}_{\text{3}}^{\text{-}}$: 56 mg N kg^-1^ | | ^15^N | | 20 | | 40 | | 84.8 | | 15.2 | | 0.254 | | 0.046 | (Wan et al., 2009) |  |
|  | 8.0 | 1.3 | 16 | 23.9 | 1.2 | 0.8 | 35.5 |  |  |  |  | 20 | | 60 | | 83.0 | | 17.0 | | 0.448 | | 0.092 |  |  |
|  | 8.0 | 1.3 | 16 | 23.9 | 1.2 | 0.8 | 35.5 | $\text{NH}_{\text{4}}^{\text{+}}$: 50 mg N kg^-1^  $\text{NO}_{\text{3}}^{\text{-}}$: 50 mg N kg^-1^，glucose C: 1% | |  |  | 20 | | 40 | | 64.7 | | 35.3 | | 0.129 | | 0.071 |  |  |
| Australia | 6.0 |  | 5 |  | 0.6 | 2.6 | 8.8 | $\text{NH}_{\text{4}}^{\text{+}}$: 100 mg N kg^-1^  $\text{NO}_{\text{3}}^{\text{-}}$: 50 mg N kg^-1^ | | ^15^N | | 25 | | 50 | | 3.30 | | 96.67 | |  | |  | (Liu et al., 2016b) |  |
|  | 7.8 |  | 1 |  | 0.8 | 1.1 | 19 |  |  |  |  | 25 | | 50 | | 76.36 | | 23.64 | |  | |  |  |  |
|  | 4.8 |  | 4 |  | 8 | 16 | 47 |  |  |  |  | 25 | | 50 | | 29.09 | | 70.90 | |  | |  |  |  |
|  | 7.0 |  | 10 |  |  | 5.1 | 10 |  |  |  |  | 25 | | 50 | | 28.74 | | 71.26 | |  | |  |  |  |
| Germany | 6.1 | 1.2 | 22 | 14.8 | 1.6 |  |  | $\text{NH}_{\text{4}}^{\text{+}}$: 10 mg N kg^-1^  $\text{NO}_{\text{3}}^{\text{-}}$: 2 mg N kg^-1^ | | ^15^N | | 15 | | 55 | | 80 | | 20 | | 0.040 | | 0.010 | (Well et al., 2008) |  |
|  | 6.1 | 1.2 | 22 | 14.8 | 1.6 |  |  | $\text{NH}_{\text{4}}^{\text{+}}$: 20 mg N kg^-1^  $\text{NO}_{\text{3}}^{\text{-}}$: 2 mg N kg^-1^ | |  |  | 15 | | 55 | | 94 | | 6 | | 0.141 | | 0.009 |  |  |
|  | 6.1 | 1.2 | 22 | 14.8 | 1.6 |  |  | $\text{NH}_{\text{4}}^{\text{+}}$: 40 mg N kg^-1^  $\text{NO}_{\text{3}}^{\text{-}}$: 2 mg N kg^-1^ | |  |  | 15 | | 55 | | 97 | | 3 | | 0.301 | | 0.009 |  |  |
|  | 6.1 | 1.2 | 22 | 14.8 | 1.6 |  |  | $\text{NH}_{\text{4}}^{\text{+}}$: 140 mg N kg^-1^  $\text{NO}_{\text{3}}^{\text{-}}$: 2 mg N kg^-1^ | |  |  | 15 | | 30 | | 83 | | 17 | | 0.050 | | 0.010 |  |  |
|  | 6.1 | 1.2 | 22 | 14.8 | 1.6 |  |  | $\text{NH}_{\text{4}}^{\text{+}}$: 140 mg N kg^-1^  $\text{NO}_{\text{3}}^{\text{-}}$: 2 mg N kg^-1^ | |  |  | 15 | | 40 | | 86 | | 14 | | 0.060 | | 0.010 |  |  |
|  | 6.1 | 1.2 | 22 | 14.8 | 1.6 |  |  | NH_4_^+^: 140 mg N kg^-1^  $\text{NO}_{\text{3}}^{\text{-}}$: 2 mg N kg^-1^ | |  |  | 15 | | 50 | | 89 | | 11 | | 0.116 | | 0.014 |  |  |
|  | 5.6 | 1.5 | 2.5 | 23.0 | 1.4 |  |  | $\text{NH}_{\text{4}}^{\text{+}}$: 40 mg N kg^-1^  $\text{NO}_{\text{3}}^{\text{-}}$: 2 mg N kg^-1^ | |  |  | 15 | | 30 | | 87 | | 13 | | 0.035 | | 0.005 |  |  |
|  | 5.6 | 1.5 | 2.5 | 23.0 | 1.4 |  |  | $\text{NH}_{\text{4}}^{\text{+}}$: 40 mg N kg^-1^  $\text{NO}_{\text{3}}^{\text{-}}$: 2 mg N kg^-1^ | |  |  | 15 | | 40 | | 92 | | 8 | | 0.037 | | 0.003 |  |  |
|  | 5.6 | 1.5 | 2.5 | 23.0 | 1.4 |  |  | $\text{NH}_{\text{4}}^{\text{+}}$: 40 mg N kg^-1^  $\text{NO}_{\text{3}}^{\text{-}}$: 2 mg N kg^-1^ | |  |  | 15 | | 50 | | 82 | | 18 | | 0.049 | | 0.011 |  |  |
| China | 8.0 | 1.2 | 17.1 | 13.0 | 1.0 |  |  | $\text{NH}_{\text{4}}^{\text{+}}$: 40 mg N kg^-1^  $\text{NO}_{\text{3}}^{\text{-}}$: 2 mg N kg^-1^ | |  |  | 15 | | 45 | | 88 | | 12 | | 1.505 | | 0.205 |  |  |
| China | 4.89 |  |  | 15 | 2.66 | 59.4 | 1.53 | $\text{NH}_{\text{4}}^{\text{+}}$: 50 mg N kg^-1^  $\text{NO}_{\text{3}}^{\text{-}}$: 50 mg N kg^-1^ | | ^15^N | | 28 | | 70 | | 20.45 | | 79.55 | |  | |  | (Liu et al., 2018) |  |
|  | 4.89 |  |  | 15 | 2.66 | 59.4 | 1.53 | $\text{NH}_{\text{4}}^{\text{+}}$: 50 mg N kg^-1^  $\text{NO}_{\text{3}}^{\text{-}}$: 50 mg N kg^-1^  glucose: 0.5 mg C kg^-1^ | |  |  | 28 | | 70 | | 12.75 | | 87.25 | |  | |  |  |  |
|  | 6.21 |  |  | 14.8 | 1.40 | 13.72 | 0.66 | $\text{NH}_{\text{4}}^{\text{+}}$: 50 mg N kg^-1^  $\text{NO}_{\text{3}}^{\text{-}}$: 50 mg N kg^-1^ | |  |  | 28 | | 70 | | 38.3 | | 61.7 | |  | |  |  |  |
|  | 6.21 |  |  | 14.8 | 1.40 | 13.72 | 0.66 | $\text{NH}_{\text{4}}^{\text{+}}$: 50 mg N kg^-1^  $\text{NO}_{\text{3}}^{\text{-}}$: 50 mg N kg^-1^  glucose: 0.5 mg C kg^-1^ | |  |  | 28 | | 70 | | 29.45 | | 70.55 | |  | |  |  |  |
| USA | 6.45 | 1.2 |  | 24.35 | 1.92 | 0.83 | 10.95 | $\text{NH}_{\text{4}}^{\text{+}}$: 7.95-34 μg N microcosm^-1^  $\text{NO}_{\text{3}}^{\text{-}}$: 148-238 μg N microcosm^-1^ | | ^15^N | | 25 | | 55 | | 56 | | 44 | | 0.129 | | 0.101 | (Hernandez-Ramirez et al., 2009) |  |
|  | 6.45 | 1.2 |  | 24.35 | 1,92 | 0.83 | 10.95 |  |  |  |  | 25 | | 90 | | 28 | | 72 | | 0.252 | | 0.648 |  |  |
| China | 7.89 |  | 7.5 | 10.93 | 1.13 | 3.07 | 22.35 | $\text{NH}_{\text{4}}^{\text{+}}$: 50 mg N kg^-1^  $\text{NO}_{\text{3}}^{\text{-}}$: 50 mg N kg^-1^ | | ^15^N | | 25 | | 40 | | 76.02 | | 23.98 | | 0.074 | | 0.023 | This study |  |
|  | 7.89 |  | 7.5 | 10.93 | 1.13 | 3.07 | 22.35 |  |  | | 25 | | 60 | | 83.90 | | 16.10 | | 0.081 | | 0.015 | |  |  |
|  | 7.89 |  | 7.5 | 10.93 | 1.13 | 3.07 | 22.35 |  |  |  | 25 | | 70 | | 33.21 | | 66.79 | | 0.349 | | 0.701 | |  |  |
|  | 7.89 |  | 7.5 | 10.93 | 1.13 | 3.07 | 22.35 |  |  |  | 25 | | 80 | | 26.49 | | 73.51 | | 1.295 | | 3.595 | |  |  |
|  | 7.89 |  | 7.5 | 10.93 | 1.13 | 3.07 | 22.35 |  |  |  | 25 | | 90 | | 19.71 | | 80.29 | | 2.491 | | 10.149 | |  |  |
|  | 7.89 |  | 7.5 | 10.93 | 1.13 | 3.07 | 22.35 |  |  |  | 25 | | 95 | | 15.71 | | 84.29 | | 1.551 | | 8.319 | |  |  |
|  | 7.89 |  | 7.5 | 10.93 | 1.13 | 3.07 | 22.35 |  |  |  | 25 | | 100 | | 11.71 | | 88.29 | | 0.327 | | 2.463 | |  |  |
|  | 7.89 |  | 7.5 | 10.93 | 1.13 | 3.07 | 22.35 |  |  |  | 25 | | 120 | | 25.82 | | 74.18 | | 0.186 | | 0.534 | |  |  |
|  | 7.92 |  | 6.71 | 19.82 | 2.11 | 2.48 | 30.49 |  |  |  | 25 | | 40 | | 85.34 | | 14.66 | | 0.137 | | 0.023 | |  |  |
|  | 7.92 |  | 6.71 | 19.82 | 2.11 | 2.48 | 30.49 |  |  |  | 25 | | 60 | | 30.69 | | 69.31 | | 0.313 | | 0.707 | |  |  |
|  | 7.92 |  | 6.71 | 19.82 | 2.11 | 2.48 | 30.49 |  |  |  | 25 | | 70 | | 26.98 | | 73.02 | | 1.576 | | 4.264 | |  |  |
|  | 7.92 |  | 6.71 | 19.82 | 2.11 | 2.48 | 30.49 |  |  |  | 25 | | 80 | | 19.75 | | 80.25 | | 2.064 | | 8.386 | |  |  |
|  | 7.92 |  | 6.71 | 19.82 | 2.11 | 2.48 | 30.49 |  |  |  | 25 | | 90 | | 13.16 | | 86.84 | | 1.709 | | 11.281 | |  |  |
|  | 7.92 |  | 6.71 | 19.82 | 2.11 | 2.48 | 30.49 |  |  |  | 25 | | 95 | | 7.68 | | 92.32 | | 1.039 | | 12.491 | |  |  |
|  | 7.92 |  | 6.71 | 19.82 | 2.11 | 2.48 | 30.49 |  |  |  | 25 | | 100 | | 12.61 | | 87.39 | | 0.414 | | 2.866 | |  |  |
|  | 7.92 |  | 6.71 | 19.82 | 2.11 | 2.48 | 30.49 |  |  |  | 25 | | 120 | | 33.74 | | 66.26 | | 0.270 | | 0.530 | |  |  |

**Table S2** The contribution of nitrification ($\text{C}_{\text{n}}$) and denitrification ($\text{C}_{\text{d}}$) to N_2_O production under different WFPS

| Soil moisture | Time | Relative contribution（%） | | | | N_2_O flux（μg N kg^-1^ h^-1^） | | | |
| --- | --- | --- | --- | --- | --- | --- | --- | --- | --- |
|  |  | SZ | | LC | | SZ | | LC | |
|  |  | $\text{C}_{\text{n}}$ | $\text{C}_{\text{d}}$ | $\text{C}_{\text{n}}$ | $\text{C}_{\text{d}}$ | N_2_On^a^ | N_2_Od^b^ | N_2_On | N_2_Od |
| 40%WFPS | h0-12 | 80.80 (2.80) | 19.20 (2.80) | 84.48(0.91) | 15.52(0.91) | 0.15(0.04) | 0.03(0.01) | 0.26(0.04) | 0.05(0.01) |
|  | h12-24 | 71.23 (10.30) | 28.77 (10.30) | 86.21(2.84) | 13.79(2.84) | 0.02(0.00) | 0.01(0.00) | 0.05(0.01) | 0.01(0.00) |
| 60%WFPS | h0-12 | 82.80 (0.67) | 17.20 (0.67) | 57.25(5.04) | 42.75(5.04) | 0.15(0.06) | 0.03(0.01) | 0.81(0.29) | 0.61(0.22) |
|  | h12-24 | 85.01 (0.42) | 14.99 (0.42) | 7.89(1.61) | 92.11(1.61) | 0.03(0.00) | 0.00(0.00) | 0.10(0.04) | 1.13(0.44) |
| 70%WFPS | h0-12 | 49.03 (4.01) | 50.97 (4.01) | 43.26(1.83) | 56.74(1.83) | 0.54(0.24) | 0.56(0.25) | 3.53(0.47) | 4.62(0.62) |
|  | h12-24 | 17.40 (6.64) | 82.60 (6.64) | 10.69(5.34) | 89.31(5.34) | 0.42(0.23) | 2.00(1.09) | 0.76(0.13) | 6.32(1.12) |
| 80%WFPS | h0-12 | 42.50 (3.04) | 57.50 (3.04) | 36.34(4.12) | 63.66(4.12) | 2.87(0.28) | 3.88(0.38) | 5.64(0.60) | 9.88(1.05) |
|  | h12-24 | 10.47 (1.37) | 89.53 (1.37) | 3.15(2.63) | 96.85(2.63) | 0.64(0.09) | 5.46(0.76) | 0.34(0.12) | 10.45(3.75) |
| 90%WFPS | h0-12 | 30.40 (2.47) | 69.60 (2.47) | 22.49(1.78) | 77.51(1.78) | 4.19(0.35) | 9.59(0.80) | 4.17(0.85) | 14.38(2.91) |
|  | h12-24 | 9.03 (3.72) | 90.97 (3.72) | 3.84(3.18) | 96.16(3.18) | 2.08(0.22) | 20.91(2.23) | 0.43(0.20) | 10.79(4.92) |
| 95%WFPS | h0-12 | 27.60 (4.59) | 72.40 (4.59) | 15.36(1.93) | 84.64(1.93) | 3.51(0.31) | 9.20(0.82) | 2.78(0.59) | 15.31(3.25) |
|  | h12-24 | 8.16 (3.16) | 91.84 (3.16) | 2.59(0.83) | 97.41(0.83) | 1.33(0.43) | 14.99(4.90) | 0.34(0.06) | 12.89(2.31) |
| 100%WFPS | h0-12 | 20.00 (2.77) | 80.00 (2.77) | 22.37(0.12) | 77.63(0.12) | 0.95(0.16) | 3.80(0.63) | 1.18(0.38) | 4.10(1.31) |
|  | h12-24 | 9.64 (10.36) | 90.36 (10.36) | 2.86(7.69) | 97.14(7.69) | 0.20(0.14) | 1.87(1.30) | 0.04(0.02) | 1.33(0.63) |
| 120%WFPS | h0-12 | 24.07 (9.05) | 75.93 (9.05) | 34.27(4.53) | 65.73(4.53) | 0.33(0.04) | 1.04(0.13) | 0.52(0.08) | 1.00(0.14) |
|  | h12-24 | 27.58 (2.32) | 72.42 (2.32) | 33.21(6.79) | 66.79(6.79) | 0.04(0.01) | 0.11(0.02) | 0.05(0.01) | 0.10(0.02) |

^a^N_2_On denotes rates of N_2_O emission from nitrification

^b^N_2_Od denotes rates of N_2_O emission from denitrification

**Table S3** Correlations between the N_2_On/N_2_Od and soil properties

|  |  | pH | BD | Clay | SOC | TN | $\text{NH}_{\text{4}}^{\text{+}}$ | $\text{NO}_{\text{3}}^{\text{-}}$ | T | WFPS |
| --- | --- | --- | --- | --- | --- | --- | --- | --- | --- | --- |
| N_2_On | R | -0.173 | 0.017 | 0.017 | 0.198 | 0.164 | 0.371* | 0.426** | 0.157 | 0.139 |
|  | *p* | 0.107 | 0.913 | 0.892 | 0.098 | 0.141 | 0.022 | 0.008 | 0.150 | 0.198 |
|  | n | 88 | 45 | 66 | 71 | 82 | 38 | 38 | 86 | 88 |
| N_2_Od | R | -0.108 | 0.034 | 0.032 | 0.167 | 0.176 | 0.376* | 0.444** | 0.200 | 0.207 |
|  | *p* | 0.317 | 0.823 | 0.801 | 0.163 | 0.113 | 0.020 | 0.005 | 0.065 | 0.053 |
|  | n | 88 | 45 | 66 | 71 | 82 | 38 | 38 | 86 | 88 |

**P*＜0.05; ***P*＜0.01





**Fig. S1** CO_2_ production rates from the SZ and LC soils under different WFPS over an incubation time of 24 hours. Different lower case letters denote significant differences between SZ and LC soil according to *t*-test, and different capital letters denote significant differences among different WFPS according to one-way analysis of variance (ANOVA) (*P*< 0.05)





**Fig. S2** ^15^N enrichments of NH_4_^+^, NO_3_^-^, and N_2_O in SZ **(A, B)** and LC **(C, D)** soils under ^15^NH_4_^+^-labeled **(A, C)** and ^15^NO_3_^-^-labeled **(B, D)** treatments





**Fig. S3** Contribution of denitrification to N_2_O production ($\text{C}_{\text{d}}$) with incubation temperature (T) across global agricultural soils. The shaded region represents the 95% confidence interval for the collected and measured data





**Fig. S4** Contribution of denitrification to N_2_O production ($\text{C}_{\text{d}}$) with WFPS across global agricultural soils. Different colors denote different pH values **(A)** and SOC contents **(B)**





**Fig. S5** Changes in the *N_2_On* with NH_4_^+^-N **(A)** and NO_3_^-^-N **(B)** and changes in the *N_2_On* with NH_4_^+^-N **(C)** and NO_3_^-^-N **(D)**. The shaded region represents the 95% confidence interval for the collected and measured data

## References

Bateman, E.J., and Baggs, E.M. (2005). Contributions of nitrification and denitrification to N_2_O emissions from soils at different water-filled pore space. *Biol. Fert. Soils* 41**,** 379-388. doi: 10.1007/s00374-005-0858-3.

Chen, Z., Ding, W., Luo, Y., Yu, H., Xu, Y., Müller, C., et al. (2014). Nitrous oxide emissions from cultivated black soil: A case study in Northeast China and global estimates using empirical model. *Glob. Biogeochem. Cycle* 28**,** 1311-1326. doi: 10.1002/2014GB004871.

Friedl, J., Scheer, C., De Rosa, D., Müller, C., Grace, P.R., and Rowlings, D.W. (2021). Sources of nitrous oxide from intensively managed pasture soils: the hole in the pipe. *Environ. Res. Lett.* 16:065004. doi: 10.1088/1748-9326/abfde7.

Hergoualc’h, K., Skiba, U., Harmand, J.M., and Oliver, R. (2007). Processes responsible for the nitrous oxide emission from a Costa Rican Andosol under a coffee agroforestry plantation. *Biol. Fert. Soils* 43**,** 787-795. doi: 10.1007/s00374-007-0168-z.

Hernandez-Ramirez, G., Brouder, S.M., Smith, D.R., Van Scoyoc, G.E., and Michalski, G. (2009). Nitrous Oxide Production in an Eastern Corn Belt Soil: Sources and Redox Range. *Soil Sci. Soc. Am. J.*  73**,** 1182-1191. doi: 10.2136/sssaj2008.0183.

Lan, T., Suter, H., Liu, R., Gao, X., and Chen, D. (2018). Nitrogen transformation rates and N_2_O producing pathways in two pasture soils. *J. soils Sediments* 18**,** 2970-2979. doi: 10.1007/s11368-018-1954-y.

Liao, X., Müller, C., Jansen-Willems, A., Luo, J., Lindsey, S., Liu, D., et al. (2021). Field-aged biochar decreased N_2_O emissions by reducing autotrophic nitrification in a sandy loam soil. *Biol. Fert. Soils* 57**,** 471-483. doi: 10.1007/s00374-021-01542-8.

Liu, H., Ding, Y., Zhang, Q., Liu, X., Xu, J., Li, Y., et al. (2018). Heterotrophic nitrification and denitrification are the main sources of nitrous oxide in two paddy soils. *Plant Soil* 445**,** 39-53. doi: 10.1007/s11104-018-3860-x.

Liu, R., Hayden, H.L., Suter, H., Hu, H., Lam, S.K., He, J., et al. (2016a). The effect of temperature and moisture on the source of N_2_O and contributions from ammonia oxidizers in an agricultural soil. *Biol. Fert. Soils* 53**,** 141-152. doi: 10.1007/s00374-016-1167-8.

Liu, R., Hu, H., Suter, H., Hayden, H.L., He, J., Mele, P., et al. (2016b). Nitrification Is a Primary Driver of Nitrous Oxide Production in Laboratory Microcosms from Different Land-Use Soils. *Front. Microbiol.* 7. doi: 10.3389/fmicb.2016.01373.

Pihlatie, M., Syvasalo, E., Simojoki, A., Esala, M., and Regina, K. (2004). Contribution of nitrification and denitrification to N_2_O production in peat, clay and loamy sand soils under different soil moisture conditions. *Nutr. Cycl. Agroecosystems* 70**,** 135-141. doi: 10.1023/B:FRES.0000048475.81211.3c.

Qin, H., Wang, D., Xing, X., Tang, Y., Wei, X., Chen, X., et al. (2021). A few key nirK- and nosZ-denitrifier taxa play a dominant role in moisture-enhanced N_2_O emissions in acidic paddy soil. *Geoderma* 385. doi: 10.1016/j.geoderma.2020.114917.

Sanz-Cobena, A., Abalos, D., Meijide, A., Sanchez-Martin, L., and Vallejo, A. (2014). Soil moisture determines the effectiveness of two urease inhibitors to decrease N_2_O emission. *Mitig. Adapt. Strateg. Glob. Chang*. 21, 1131-1144. doi: 10.1007/s11027-014-9548-5.

Thilakarathna, S.K., and Hernandez-Ramirez, G. (2021). Primings of soil organic matter and denitrification mediate the effects of moisture on nitrous oxide production. *Soil Biol. Biochem.* 155: 108166. doi: 10.1016/j.soilbio.2021.108166.

Wan, Y., Ju, X., Ingwersen, J., Schwarz, U., Stange, C.F., Zhang, F., et al. (2009). Gross Nitrogen Transformations and Related Nitrous Oxide Emissions in an Intensively Used Calcareous Soil. *Soil Sci. Soc. Am. J.* 73**,** 102-112. doi: 10.2136/sssaj2007.0419.

Well, R., Flessa, H., Xing, L., Xiaotang, J., and Römheld, V. (2008). Isotopologue ratios of N_2_O emitted from microcosms with NH_4_^+^ fertilized arable soils under conditions favoring nitrification. *Soil Biol. Biochem.* 40**,** 2416-2426. doi: 10.1016/j.soilbio.2008.06.003.

Zheng, Q., Ding, J., Li, Y., Lin, W., Xu, C., Li, Q., et al. (2017). The Effects of Soil Water Content on N_2_O Emissions and Isotopic Signature of Nitrification and Denitrification. *Scientia Agricultura Sinica* 50**,** 4747-4758.
